# Free-Living Nematodes Together With Associated Microbes Play an Essential Role in Apple Replant Disease

**DOI:** 10.3389/fpls.2018.01666

**Published:** 2018-11-16

**Authors:** Xorla Kanfra, Benye Liu, Ludger Beerhues, Søren J. Sørensen, Holger Heuer

**Affiliations:** ^1^Department of Epidemiology and Pathogen Diagnostics, Julius Kühn-Institut - Federal Research Centre for Cultivated Plants, Braunschweig, Germany; ^2^Institute of Pharmaceutical Biology, Technische Universität Braunschweig, Braunschweig, Germany; ^3^Section of Microbiology, Department of Biology, University of Copenhagen, Copenhagen, Denmark

**Keywords:** apple replant disease, nematodes, phytoalexins, *Malus domestica*, nematode-microbe interaction, soil microbiota

## Abstract

Apple replant disease (ARD) is a severe problem in apple production worldwide. It is caused by a complex of soil biota, leading to small discolorated roots, as well as increased biosynthesis of phytoalexins, total phenolic compounds and antioxidants. We sampled soil from randomized field plots with either apple trees affected by ARD, which were five times replanted every second year, or with healthy trees growing in plots, which had a grass cover during this period. We investigated the contribution of nematodes to ARD by dissecting the soil biota from plots infested with ARD and non-infested control plots into a nematode and a microbe fraction. Nematode communities significantly differed between ARD and control soil as revealed by high-throughput sequencing of 18S rRNA genes. Plant-parasitic nematodes were too low in abundance to explain root damage, and did not significantly differ between ARD and control soil. Their separate and synergistic effect on ARD symptoms of susceptible M26 apple rootstocks was analyzed 4 and 8 weeks after inoculation in three greenhouse experiments. Inoculants were either nematodes from ARD plots (N_ARD_), N_ARD_ plus microbes from ARD plots (M_ARD_), N_ARD_ plus microbes from control plots (M_Con_), nematodes from control plots N_Con_ plus M_ARD_, N_Con_ plus M_Con_, M_ARD_, or M_Con_, or non-inoculated control. In all three experiments, the combination N_ARD_ plus M_ARD_ had the strongest adverse effect on the plants, with respect to growth parameters of shoots and roots, total phenolic compounds and phytoalexins in roots, and antioxidants in leaves. N_ARD_ also induced ARD but less than N_ARD_ plus M_ARD_. N_ARD_ plus M_Con_ had delayed effects on the plants compared to N_ARD_ plus M_ARD_, suggesting that detrimental nematode-microbe interactions built up with time. Effects of M_ARD_ or N_Con_ plus M_ARD_ were minor or not distinguishable from those of M_Con_ or non-inoculated control. Overall, the source of the inoculated nematodes -ARD or control soil- and the interaction between ARD nematodes and microbes were highly significant factors determining ARD. In conclusion, exploring the associations of nematodes and microbes in ARD soils will give the chance to unravel the etiology of ARD.

## Introduction

After the repetitive planting of apple on the same site for extended periods of time, the soil loses its capacity to support plant growth. Young apple trees planted on these soils exhibit severe growth reduction (Mai and Abawi, 1981). This problem has been named apple replant disease (ARD) (Ross and Crowe, 1973). Symptoms associated with poor tree growth are characterized by reduced shoot and root growth, smaller leaves, browning of roots (Mazzola and Manici, 2012), as well as accumulation of phenolic compounds in roots and antioxidants in leaves (Henfrey et al., 2015). The poor plant growth is typically persistent, and the disease does not spread through sites (Klaus, 1939). The cause of ARD is unclear although numerous studies have focused on it for decades (Mazzola and Manici, 2012; Winkelmann et al., 2019). The significantly improved growth of apple plants after soil pasteurization or fumigation gives substantial evidence that the disease is caused by biotic factors (Mai and Abawi, 1978; Hoestra, 1994; Yim et al., 2013).

Nematodes occupy a key position in the soil food web and are suitable indicators of soil quality (Yeates et al., 1993). They preferably move toward the root zone for resources. A few of the plant-parasitic species invade roots resulting in crop losses, and therefore are well studied (Jones et al., 2013). In contrast, the interactions of free-living nematodes including bacteria, fungi and root feeders with plants have been hardly studied. The interaction of free-living nematodes and microbes could affect higher organisms as in the case of the entomopathogenic nematodes. Specific microbiomes associated with the surface of nematodes were reported, which were distinct from the microbiomes of the surrounding soil with respect to most abundant species (Adam et al., 2014; Elhady et al., 2017). One of the associations of nematodes with toxin-producing bacteria has been well studied (Riley and Reardon, 1995). Fungivorous or bacterivorous nematodes have feeding preferences and may thereby be associated with specific microbiomes. Specific associations of microbes with nematodes may also be the basis for the many soilborne disease complexes comprised of nematodes and microbes that were reported (Back et al., 2002; Morris et al., 2016). Root lesion nematodes (*Pratylenchus* spp.) cause synergistic damage to diverse apple hosts by acting in combination with species of *Pythium, Phytophthora, Cylindrocarpon, Fusarium*, and *Rhizoctonia* (Utkhede et al., 1992; Mazzola, 1998; Isutsa and Merwin, 2014). *Cylindrocarpon*-like fungi*, Rhizoctonia, Fusarium*, as well as the oomycetes *Pythium* and *Phytophthora* frequently coincided with ARD (Mazzola, 1998; Tewoldemedhin et al., 2011; Manici et al., 2017, 2018). Recently, a shift in the soil or rhizosphere microbiome was observed in ARD affected soils compared to healthy soils (Hewavitharana and Mazzola, 2016; Nicola et al., 2017, 2018) and could be linked to a possible cause of ARD although it is not clear if the missing or additional taxa observed in the shift could be the cause of ARD (Jiang et al., 2017).

The establishment of the pathogenicity of these agents is, however, not consistent partly because they vary greatly in their aggressiveness, or biotic and abiotic factors mediate their functions (Manici et al., 2015). Previous studies have shown that growth was not affected when single isolates of *Pythium ultimum*, or *Rhizoctonia solani*, nor in combination with *Fusarium solani* were inoculated on apple rootstocks (Mazzola, 1998). Some studies suggested a role of plant-parasitic nematodes in the disease development citing notably uneven distribution pattern of *Pratylenchus penetrans* in apple orchards (Mai and Abawi, 1978; Jaffee et al., 1982; Mai et al., 1994). However, the role of plant-parasitic nematodes in the disease complex varies among regions and in some cases populations were either below the damage threshold or not observed at all (Hoestra and Oostenbrink, 1962). None of the above-mentioned factors were consistently correlated with ARD in affected orchards, and might just be opportunistic infections of apple plants that were etiolated by ARD. Indeed, a recent comprehensive reanalysis of studies on ARD suggested more complex disease causes than single pathogens and favored a multifactorial origin of ARD, i.e., the multivariate ecological ARD hypothesis (Nicola et al., 2018). Evidence points to a disease complex of soil biota being responsible for ARD (Tewoldemedhin et al., 2011; Mazzola and Manici, 2012). Some observations indicated that nematodes are involved in such a disease complex. Soil treatments that primarily affect nematodes, as heating to 50°C or Brassica seed meal amendments reduced disease symptoms of ARD (Mazzola, 1998; Yim et al., 2013, 2016; Hu et al., 2016). However, these treatments shifted the microbiome structure (Omirou et al., 2011; Mazzola et al., 2015; Hu et al., 2016), making conclusions about the contribution of different soil biota to ARD difficult. Previous research focused on plant-parasitic nematodes, mainly *P. penetrans*, while the involvement of free-living nematodes in ARD has not been investigated, and the potential contribution of their associated microbiome has been ignored.

In this study, we extracted and separated the microbial fraction and the nematode fraction from soil and investigated their discrete or synergistic effect on ARD development of susceptible Malling 26 (M26) apple rootstocks. ARD and control soils were obtained from an experimental field in complete randomized design. In plots with replanted apple rootstocks, the plants showed severe ARD symptoms (Mahnkopp et al., 2018). Control soil was obtained from former grass plots interspersed in the same field, which were planted with apple rootstocks for the first time. The objectives were to test in a controlled experimental system, (i) whether the microbial community from ARD soil induce the disease symptoms of ARD in roots of apple rootstocks, in contrast to the microbial community from control soil; (ii) whether the nematode community from ARD soil in conjunction with the microbes enhances ARD in contrast to nematodes from the control soil. We compared the species composition of the nematode communities from ARD and control plots to investigate whether structural differences coincide with differences in their contribution to ARD, and to confirm that plant-parasitic nematodes did not affect the apple rootstocks in our test system.

## Materials and Methods

### Soil Sample Collection

Apple replant disease soils were obtained from a field in the Pinneberg area, Germany (53°41′57.1″N 9°40′59.4″E). Since 2009, rootstock of the cultivar ‘Bittenfelder Sämling’ was planted repeatedly in a 2 years cycle resulting in a 5th replanted generation in 2017 (Mahnkopp et al., 2018). The site had four plots randomly arranged with plants showing severe ARD symptoms (referred to as ARD plots). The ARD plots were interspersed with four plots previously covered with grass that have been planted with apple rootstocks for the first time (referred to as control plots). From each ARD and control plot, soil was sampled around roots of three individual plants in a zig-zag pattern at a depth of 0–30 cm. Thereby, 24 soil samples (250 ml each) were collected for the initial assessment of nematode composition before the incubation studies. The rest of the soil samples from the ARD or control soils were pooled together to form composite samples for total soil nematode and microbiome extraction. These soil samples were gently sieved through a 5 mm mesh to homogenize the soil and to remove visible soil organisms, stones, and plant debris. They were then stored at 4°C for 2 weeks before the pot experiments.

### Soil Nematode and Microbiome Extraction

Nematodes (plant-parasitic and free-living) were extracted from 250 ml portions of ARD or control soil by centrifugal floatation using MgSO_4_ at 1.18 specific density (Hooper et al., 2005). Nematodes were collected on a 20 μm sieve and thoroughly washed with sterile water. To extract nematodes from the roots, a modified Baermann funnel technique was used (Barker, 1985). Roots were carefully washed, cut into about 1 cm sized pieces, wrapped in a tissue cloth and spread on a mesh (1 mm aperture). This was then placed on a Baermann funnel filled with water to about 5 mm above the mesh. Nematodes were collected from the stem of the funnel after 2 weeks of incubation. Nematodes were transferred to a glass beaker and counted on a counting slide under an Olympus SZX12 stereomicroscope at 40×–80× magnification (Olympus, Hamburg, Germany) to adjust the suspension to 100–125 individuals per ml for the inoculation of pots.

To prepare the microbial inoculants, 100 g of soil was added to 100 g of 0.5 mm glass beads (Zolla et al., 2013). This was suspended in 900 ml sterile saline and shaken for 1 hr with an orbital shaker (200 rpm). After settling for 1 h, the supernatant was centrifuged (1000 × *g*, 5 min) to pellet soil particles. The resulting supernatant containing suspended soil microbes was passed through a 5 μm sieve in order to remove nematodes. Microbes were pelleted at 4000 rpm (3470 × *g*) for 30 min at 4°C, and resuspended in sterile tap water for inoculations in pot experiments. Each plant received 20 ml of microbial suspension, corresponding to microbes of 10 g of soil, per 1000 ml sterile sand-perlite mix.

#### Community Analysis of the Nematode Inocula

Total community DNA from nematodes was extracted using the FastPrep FP120 bead beating system and FastDNA SPIN Kit for Soil (MP Biomedicals, Santa Ana, CA, United States) as described by the manufacturer. The DNA was purified with GENECLEAN SPIN Kit (MP Biomedicals) according to the manufacturer’s instructions. For the nematode species composition analysis, primers G18S4F and G18S4-22R were used to amplify approximately 345 bp of the 18S rRNA gene (Blaxter et al., 1998). PCR of 25 μl contained 2.5 μl of 10× GoTaq buffer (Promega, Mannheim, Germany), 3.75 μl 25 mM MgCl_2,_ 2.5 μl 2 mM dNTP (each), 0.5 μl of each primer (10 μM), 1.25 μl of 2 mg/ml BSA, 2 μl of 50% acetamid, 0.2 μl 5 U/μl GoTaq DNA polymerase (Promega), ca. 2 ng DNA. The following PCR cycler conditions was used: initial denaturation of 5 min at 94°C, 27 cycles of (94°C for 45 s; 54°C for 30 s; 72°C for 1 min) and a final extension of 5 min at 72°C. The resulting PCR product was purified using High Pure PCR Purification kit (Roche Diagnostics GmbH) following the manufacturers instruction. Barcoded amplicon sequencing of the 18S rRNA genes was done by 2 × 250 bp paired-end high-throughput sequencing on an Illumina MiSeq platform (Illumina, San Diego, CA, United States).

### Greenhouse Assay

Perlite and sand mix (1:4) was sterilized by autoclaving (20 min at 121°C, 3 pulses). *In vitro* propagated, acclimatized apple M26 rootstocks (14 days old), were received from Dr. Traud Winkelmann (Leibniz Universität Hannover, Germany) and used as susceptible genotypes (Yim et al., 2013). Plants were transferred into pots containing 1 l of the sterile sand-perlite mix and grown for 7 days before nematodes and/or microbes were inoculated. Inoculation of nematodes and/or microbes was done by digging 5 cm deep 1 cm wide holes in 2 cm distance around the shoot to enable the equal distributing of 40 ml of nematode suspension and/or 20 ml of microbial suspension. The fertilizers NPK (+Mg) [15:10:15(+2)] (0.5 g/l) and 36% Calcium (2g/l) were applied weekly. Plants were watered by hand as required. The greenhouse conditions were 22 ± 2.5°C, 60 ± 8.7% relative humidity and a 16 h photoperiod. In each experiment, pots were placed in a randomized complete block design in the greenhouse. Pots were sampled 4 and 8 weeks after inoculation. Shoot length, shoot fresh mass, leaf count, leaf fresh mass, root fresh mass were determined. Leaf dry mass and root dry mass were only determined 8 weeks after inoculation. Additionally, 250 mg of fresh leaves or roots were frozen in liquid nitrogen and subsequently frozen at -80°C for analysis of antioxidant and total phenolic compounds.

In the first experiment, we investigated whether nematodes from ARD soil increased disease symptoms of apple plants. For this purpose, 40 ml of microbial inocula from ARD soil (M_ARD_) in combination with 20 ml nematode suspension from ARD soil (N_ARD_) were inoculated in the root zone of potted apple plants. Potted plants also received only M_ARD_. Untreated plants, which were not inoculated, served as control treatments (U). There were 20 replicates per treatment making a total of 40 treated plants and 20 untreated plants.

In the second experiment, we investigated whether N_ARD_ induced stronger disease symptoms than nematodes from control soil (N_Con_). We hypothesized that the M_ARD_ affects the plants more than the microbial community from control soil (M_Con_), and N_ARD_ enhance this negative effect more than N_Con_. There were seven treatments in total for this experiment. This included N_ARD_+M_ARD_, N_ARD_ + M_Con_, N_Con_ + M_ARD_, N_Con_ + M_Con_, M_ARD_, M_Con_, and U, with 20 replicates per treatment.

In the third experiment, we tested whether N_ARD_ alone are enough to induce ARD symptoms of apple plants. There were four treatments including N_ARD_, N_ARD_+M_ARD_, M_ARD_ and U with 20 replicates per treatment, making a total of 80 pots. In addition to the other parameters, phytoalexins in roots were analyzed.

### Analysis of Antioxidants, Total Phenolics, and Phytoalexins

To determine if N_ARD_+M_ARD_ induce physiological plant response in apple plants, the concentrations of antioxidants in the leaves and total phenolic and phytoalexin compounds in the roots were determined as indicators of ARD. To achieve this, shock-frozen leaf or root (0.25 g) was disrupted and homogenized using a tissue lyser II (QIAGEN, Germany). Homogenization was achieved with a 3 mm glass bead at 30 Hz for 3 min. The milled samples were taken up with 0.7 ml ice-cold ethanol (99% vol/vol) and homogenized for 5 min. Cooled samples were centrifuged (13000 *g* for 10 min at room temperature). The supernatant was transferred to a fresh 2 ml microtube and stored at -20°C until subsequent analysis.

The antioxidant concentration in the leaves was measured using the ABTS (3-ethyl benzo- thiazoline-6-sulfonate) assay at 660 nm as described previously (Erel, 2004). Antioxidant concentration was measured against the standard trolox (6-Hydroxy-2,5,7,8-tetramethylchromane-2-carboxylic acid) (Sigma-Aldrich, Germany) and concentrations expressed as mg Trolox equivalent per mg fresh mass. Total phenolic concentrations in the roots were measured using the Folin Ciocalteau reagent at 765 nm using a spectrophotometer (Ainsworth and Gillespie, 2007). Total phenolic concentration was calculated against a gallic acid standard and was expressed in mg gallic acid equivalents per g fresh mass. Analysis of phytoalexins was carried out by a gas chromatography-mass spectrophotometry as described previously (Hüttner et al., 2010).

### Data Analysis

The 18S rRNA sequence demultiplexing was done using the MiSeq Controller Software and diversity spacers were trimmed using Biopieces^1^. Overlapping regions within paired-end reads were aligned to generate “contigs” and primers removed from both ends of the sequences by PANDAseq using default settings (Masella et al., 2012). Taxonomic affiliations were assigned by BlastN against the Silva SSU 128 database (Quast et al., 2013). De-replication, singleton removal and clustering of sequences to operational taxonomic units (OTUs, >99% similarity) were performed using BLAST Parser (Antweiler et al., 2017) implemented in a Galaxy workflow (Cock et al., 2013). The multivariate analyses on the OTU abundance table were carried out with the R software version R3.1.3^2^ with the packages vegan (Oksanen et al., 2015), EdgeR (Robinson et al., 2010), and LabDSV (Roberts, 2016). Significance of differences in nematode communities were tested by PERMANOVA (Anderson, 2001) based on Bray-Curtis dissimilarities using 10,000 permutations. Nematode community composition was visualized on untransformed data by non-metric multi-dimensional scaling (NMDS) 20 to 100 times randomly computed based on Bray-Curtis similarities using default settings (McCune et al., 2002). To test for significantly different abundant OTUs (likelihood ratio test under negative binomial distribution and generalized linear models, false discovery rate -corrected *P* < 0.05) between ARD and control plots, normalization of read count data was performed as recommended by the developers (EdgeR) and only OTUs present in at least three samples were considered. Statistical analysis of variance using generalized linear models and principal component analysis were done using SAS 9.4 (SAS Institute Inc., Cary, NC, United States).

## Results

### Nematode Inoculants From ARD and Control Soil Differ in Species Composition

**FIGURE 1:**
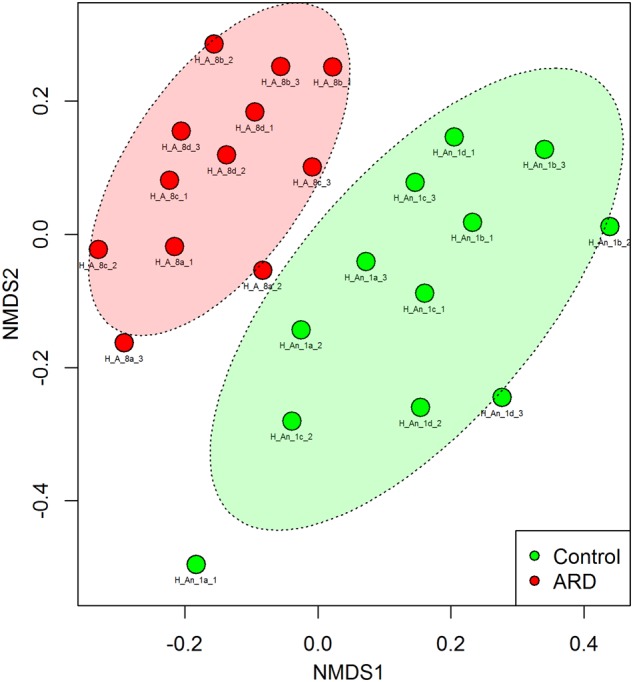
Non-metric multidimensional scaling (NMDS) based on Bray-Curtis similarities of nematode community structures from field plots replanted five times with apple or virgin control plots planted one time with apple, analyzed by high-throughput 18S rDNA amplicon sequencing from total nematode community DNA. PERMANOVA revealed a significant difference in the overall nematode community structure between ARD and control soils (*P* < 0.001, Stress = 0.246). The site had four plots per treatment randomly arranged. From each plot, soil was sampled around roots of three individual plants for extraction of nematodes to obtain total community DNA. Labels: site Heidgraben (H), replanted apple plot (A_8) or new apple plot (An_1), replicate plot (a, b, c, and d), soil subsample from individual root system (1, 2, and 3).

Before the start of our experiment, we investigated the nematode species composition from the five-time apple replanted field plots with high disease incidence (ARD) and compared it to the uncultivated grass plots (control) that were planted with apple for the first time. Twelve soil samples from four blocks each were taken from the ARD plots or control plots. The 18S rRNA gene amplicon sequence data revealed that the nematode species composition in the two soils was significantly different (PERMANOVA; *P* < 0.001). Nematode communities from ARD plots clearly clustered apart from controls in the NMDS plot (Figure 1). We identified the nematodes that were enriched in response to ARD at least to the genus level. Some nematode species were significantly sensitive to ARD (false discovery rate <0.05) (Supplementary Table S3). The genera *Acrobeloides* and *Cephalenchus* showed a significant increase in abundance in ARD plots. *Steinernema* was largely overrepresented in control plots. Notably, the abundance of the plant parasitic nematodes was not significantly different between ARD and control plots. Also the relative abundance of the root-lesion nematode *P. penetrans* in the two soils was not significantly different. Extraction of nematodes from the roots of the diseased apple plants did not yield any endogenous *P. penetrans*. The typical symptoms, which include root lesions or lack of feeder roots, were not observed with the diseased roots. Supplementary Table S4 provides the total nematode species composition used as inoculants in our experiments.

### Nematodes From ARD Soil Increased Disease Symptoms of Apple Plants

**FIGURE 2:**
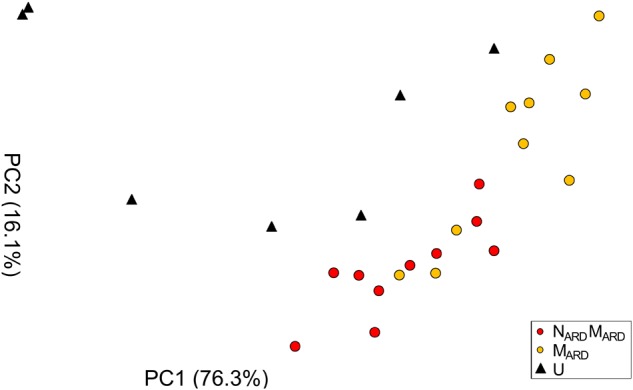
Apple plant growth affected by nematodes from ARD plots (N_ARD_) and microbes from ARD plots (M_ARD_), in comparison to the non-inoculated control (U). The first two principal components (PC1 and PC2) extracted from the parameters root weight, shoot weight, shoot length, weight of leaves, and number of leaves as determined 8 weeks after inoculation were plotted (the percentage of variance explained by PC1 and PC2 is shown in brackets).

**FIGURE 3:**
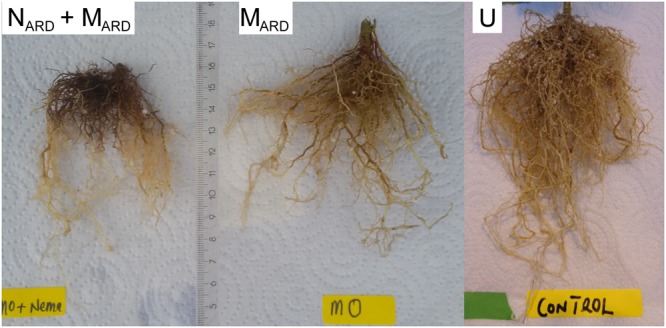
Roots of apple rootstocks that were affected by microbes extracted from ARD soil (M_ARD_), or M_ARD_ in combination with nematodes extracted from ARD soil (N_ARD_+M_ARD_) compared to roots of the non-inoculated control (U).

**FIGURE 4:**
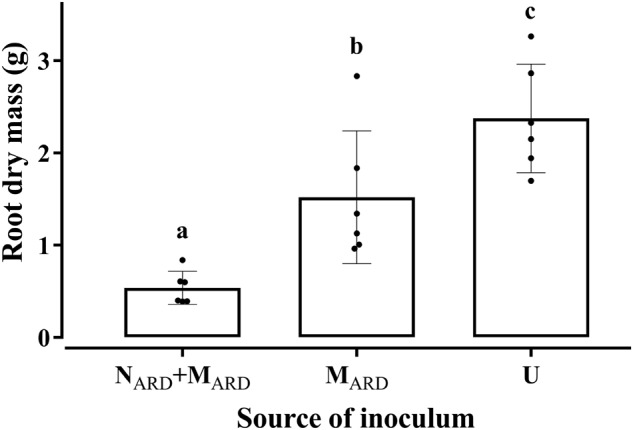
Root dry mass of apple rootstocks in response to inocula of nematodes plus microbes from ARD soil (N_ARD_+M_ARD_), or only microbes (M_ARD_), in comparison to the non-inoculated control (U). Root dry mass was determined 8 weeks after inoculation. Nematodes and microbes were from soil of an apple orchard where the plants were highly affected by apple replant disease. Mean ± SD (*n* = 6), different letters indicate significant differences revealed by Tukey’s test.

In the first experiment, we investigated whether the microbial community from ARD soil (M_ARD_) impaired growth of apple plants in our system, and whether nematodes from ARD soil (N_ARD_) added to this effect. Soil microbiota was extracted from soil of five times replanted field plots, where apple plants showed severe ARD symptoms. Nematodes were removed from the microbial suspension by sieving (<5 μm). The nematode fraction was obtained by floatation on a dense MgCl_2_ solution and sieving (>20 μm). Apple rootstocks growing in a sterile pot system were inoculated with M_ARD_ or N_ARD_+M_ARD_, or left untreated. Plants were sampled after 4 and 8 weeks. All treatments differed significantly among each other in their effect on plant growth, as revealed by MANOVA of shoot length, shoot weight, leaf number, leaf weight, and root weight. In a principal component analysis of these plant growth parameters 8 weeks after inoculation, the first two principal components, which explained together 92% of the variance, clustered plants according to the treatment (Figure 2). Root systems from treatments M_ARD_ and N_ARD_+M_ARD_ were reduced in volume compared to untreated plants and had the brown spots typical for ARD (Figure 3). Root lesions, leaf spots, necrosis, lack of feeder roots or other signs of pathogen attack were not observed. In addition, nematode extraction from roots did not reveal any endoparasitic nematodes. The root weight of M_ARD_ treated plants was significantly reduced compared to untreated plants, suggesting that the microbes induced symptoms of ARD in our system (Figure 4). The addition of nematodes significantly enhanced these effects. Shoot length of the M_ARD_+N_ARD_ treated plants was reduced 4 and 8 weeks after inoculation compared to plants inoculated with M_ARD_ only, leaf and root dry weight after 8 weeks (Supplementary Table S1). Affected plants had more and smaller leaves.

**FIGURE 5:**
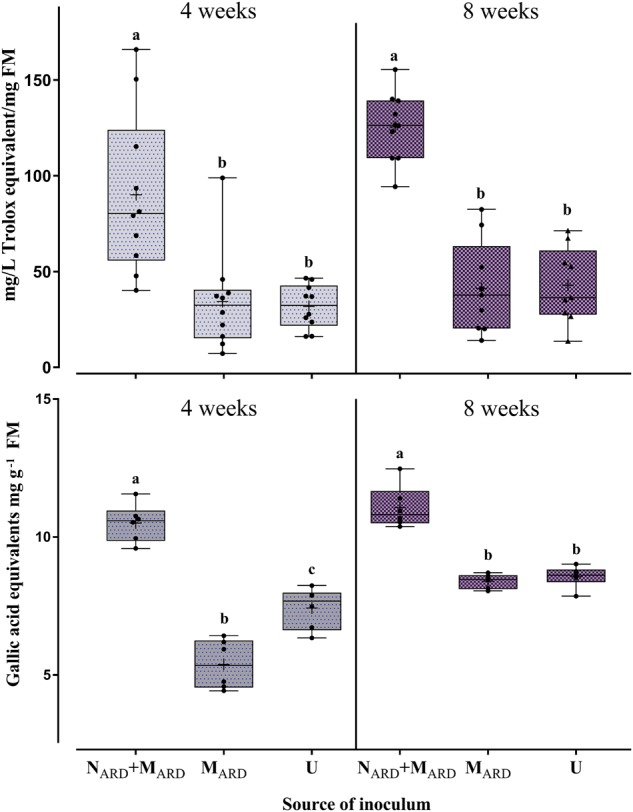
Physiological response of apple rootstocks to inocula of nematodes plus microbes from ARD soil (N_ARD_+M_ARD_), or only microbes (M_ARD_), in comparison to the non-inoculated control (U). Mean ± SD of concentrations of antioxidant compounds in leaves (upper panel, *n* = 10), and of total phenolic compounds in roots (lower panel, *n* = 6) are shown. Different letters indicate significant differences revealed by Tukey’s test.

The concentrations of total phenolic compounds in the roots and antioxidants in the leaves were determined as indicators of the physiological response to ARD. Four and eight weeks after inoculation, plants challenged by N_ARD_+M_ARD_ accumulated significantly higher concentrations of phenolics and antioxidants compared to plants inoculated with M_ARD_ only, or non-inoculated controls (Figure 5). The treatment with microbes alone did not increase the stress indicators compared to the non-inoculated control.

### Nematodes From ARD Soil Induced Stronger Disease Symptoms Than Nematodes From Control Soil

**FIGURE 6:**
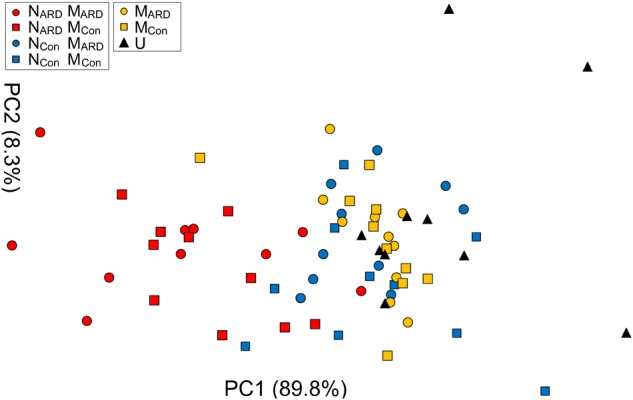
Apple plant growth affected by nematodes from ARD plots (N_ARD_) or control plots (N_Con_), and microbes from ARD plots (M_ARD_) or control plots (M_Con_), and non-inoculated control (U). The first two principal components extracted from the parameters root weight, shoot weight, shoot length, weight of leaves, and number of leaves 8 weeks after inoculation were plotted (PC1 and PC2 with the explained percentage of variance, respectively).

At the sampled field site, plots with replanted apple plants were interspersed with control plots covered with grass. In the second experiment, we compared the effects of combinations of nematodes and microbes from ARD plots and control plots on apple plants. We hypothesized that the microbial community from ARD soil (M_ARD_) affects the plants more than the microbial community from control soil (M_Con_), and that nematodes from ARD soil (N_ARD_) enhance this negative effect more than the nematodes from control soil (N_Con_). Principal component analysis of the plant growth parameters 8 weeks after inoculation revealed a cluster of the two treatments with N_ARD_ and a cluster of the treatments without N_ARD_ (Figure 6). Treatments were mainly separated on PC1 that explained 90% of the variance. The origin of the inoculated nematodes from ARD or control plots had a significant effect on plant growth, while an effect of the different soil microbiomes was not detectable (Table 1). When comparing the inoculated treatments that did not receive N_ARD_, then neither the origin of the microbiomes was a significant factor nor did the inoculation of nematodes from control plots significantly affect plant growth (Table 1). The non-inoculated control was excluded from these analyses because the variation of plant growth within this treatment was much higher than for the other treatments.

**Table 1:** Analysis of variance of plant growth parameters as represented by the first principal component (PC1) as affected by the soil from which inoculated nematodes or microbes originated.

	Analyzed levels of class variables (source of nematodes/microbes)	
Effect on dependent variable PC1	Nematodes: ARD, control Microbes: ARD, control Treatments included^a^: N_ARD_+M_ARD_, N_ARD_+M_Con_, N_Con_+M_ARD_, N_Con_+M_Con_	Nematodes: control, non-inoculated Microbes: ARD, control Treatments included^a^: N_Con_+M_ARD_, M_ARD_, N_Con_+M_Con_, M_Con_
Source of nematodes	*P* < 0.0001	*P* = 0.89
Source of microbes	*P* = 0.38	*P* = 0.80
Interaction	*P* = 0.69	*P* = 0.57
	93% variance in PC1	75% variance in PC1

*^*a*^Inoculants: N_*ARD*_, nematodes from plots with apple replant disease (ARD) causing soil; N_*Con*_, nematodes from control plots; M_*ARD*_, microbes from ARD plots; M_*Con*_, microbes from control plots.*

**FIGURE 7:**
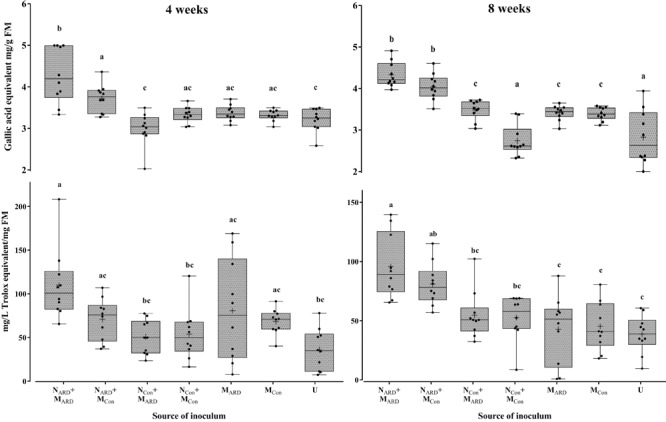
Total phenolic compounds in roots (upper panel) and antioxidants in leaves (lower panel) of apple rootstocks after inoculation with nematodes (N) and/or microbes (M) from replanted soil (ARD) or control plots (Con), 4 and 8 weeks after inoculation. Mean ± SD (*n* = 10), different letters indicate significant differences revealed by Tukey’s test.

Four weeks after inoculation, plants inoculated with N_ARD_ already showed a trend for reduced growth, which became significant 8 weeks after inoculation for aboveground parts (Supplementary Table S2). Roots of N_ARD_ treated plants were clearly affected. The root weight was significantly reduced compared to treatments with only M_ARD_, M_Con_, or non-inoculated, while the reduction compared to treatments with N_Con_ was statistically not significant due to high variation among replicates. Plants inoculated with nematodes from control plots (N_Con_+M_ARD_, N_Con_+M_Con_) did not significantly differ in plant growth compared to treatments without nematodes (M_ARD_, M_Con_, or non-inoculated control). The concentrations of total phenolic compounds in roots and antioxidants in leaves were highest in plants challenged with nematodes from ARD soil, i.e., treatments N_ARD_+M_ARD_ and N_ARD_+M_Con_ (Figure 7). The added microbes from ARD soil significantly enhanced the effect of N_ARD_ on total phenolics compared to added microbes from control soil already after 4 weeks, and antioxidants followed this trend. Eight weeks after inoculation, both treatments N_ARD_+M_ARD_ and N_ARD_+M_Con_ became more similar, and significantly differed in total phenolics from all other treatments without N_ARD_. Overall, the factors nematodes (from ARD or control) and microbes (from ARD or control) both showed a significant effect on total phenolic compounds and antioxidants when applying generalized linear models (*P* < 0.0001 and *P* = 0.0003, respectively).

### Nematodes From ARD Soil Alone Induced Disease Symptoms of Apple Plants

**FIGURE 8:**
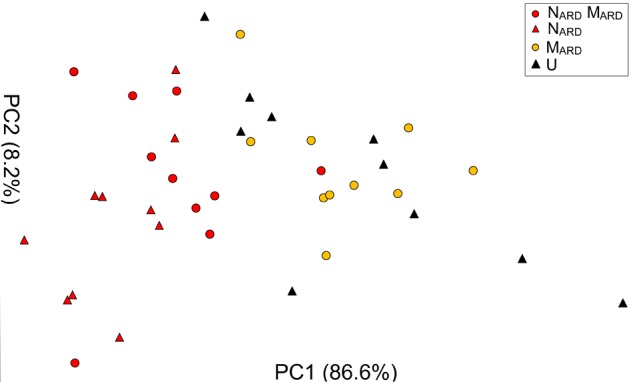
Apple plant growth affected by nematodes from ARD plots (N_ARD_) alone, or in combination with microbes from ARD plots (N_ARD_+M_ARD_,) or only microbes from ARD plots (M_ARD_), and non-inoculated control (U). The first two principal components extracted from the parameters root weight, shoot weight, shoot length, weight of leaves, and number of leaves 8 weeks after inoculation were plotted (PC1 and PC2 with the explained percentage of variance, respectively).

The previous experiments suggested that the nematodes play a pivotal role in the disease complex. In the third experiment, we tested whether N_ARD_ alone are enough to induce ARD symptoms of apple rootstocks. We compared the effect of N_ARD_ on plant growth and stress response to those of N_ARD_+M_ARD_, M_ARD_, and non-inoculated control. Notably, N_ARD_ included the microbiomes associated with the bodies of the nematodes. Principal component analysis of the plant growth parameters 8 weeks after inoculation revealed a cluster of the two treatments with N_ARD_ and a cluster of the treatments without N_ARD_ (Figure 8). Treatments were mainly separated on PC1 that explained 87% of the variance. In a generalized linear model analysis, inoculation of nematodes from ARD had a significant effect on PC1 (*P* < 0.0001), but also the effect of the microbes was significant (*P* = 0.049). The non-inoculated control was excluded from this analysis because the variation of plant growth was much higher than within the other treatments. Length and weight of shoots, number and weight of leaves, and weight of roots did not significantly differ among plants of treatments N_ARD_+M_ARD_ and N_ARD_, neither 4 nor 8 weeks after inoculation (Table 2). After 8 weeks, plants inoculated with N_ARD_+M_ARD_ or N_ARD_ significantly differed to both treatments M_ARD_ and non-inoculated control in all analyzed plant parameters. Non-inoculated plants had greater weights of stems, leaves and roots after 4 weeks, which was not significant anymore but still a trend after 8 weeks.

**Table 2:** Vegetative growth of apple rootstocks in pots inoculated with only nematodes from ARD soil (N_ARD_) or in combination with microbes from ARD plots (N_ARD_+M_ARD_,) or only microbes from ARD plots (M_ARD_), and non-inoculated control (U).

Inoculants	Weeks after inoculation	Shoot length [cm]	Shoot FM [g]	Leaf count	Leaf FM [g]	Root FM [g]
N_ARD_+M_ARD_	4	5.0 ± 1.3a	2.0 ± 0.58a	15 ± 2.2	1.5 ± 0.2a	3.6 ± 1.3a
	8	12.4 ± 2.9a	4.5 ± 0.5a	20 ± 2.5a	3.2 ± 0.7a	4.6 ± 0.5a
N_ARD_	4	5.5 ± 0.9a	2.1 ± 0.3a	15 ± 1.8a	1.4 ± 0.1a	3.5 ± 0.7a
	8	10.8 ± 1.5a	4.4 ± 0.6a	18 ± 2.4a	3.0 ± 0.5a	4.4 ± 1.0a
M_ARD_	4	10.2 ± 0.2b	3.4 ± 0.4b	19 ± 1.7b	2.2 ± 0.3a	4.8 ± 1.4a
	8	18.2 ± 2.9b	6.6 ± 1.4b	24 ± 1.6b	4.7 ± 0.7b	6.1 ± 0.6b
Non-inoculated	4	10.5 ± 2.5b	4.3 ± 0.6c	21 ± 4.6b	3.4 ± 1.2b	6.6 ± 1.1b
	8	18.8 ± 5.8b	7.0 ± 1.4b	24 ± 2.2b	5.1 ± 0.6b	6.7 ± 0.9b

*Mean ± SD (*n* = 10), different letters indicate significant differences revealed by Tukey’s test.*

**FIGURE 9:**
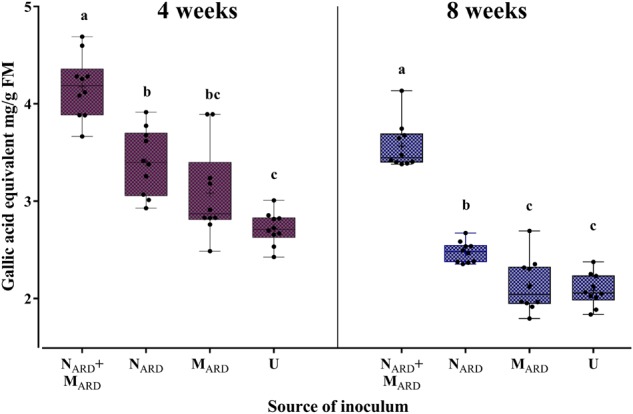
Total phenolic compounds in roots of apple rootstocks 4 or 8 weeks after inoculation with the nematodes and microbes from ARD soil (N_ARD_+M_ARD_), only nematodes from ARD soil (N_ARD_), or only microbes from ARD soil (M_ARD_), and non-inoculated control (U). Mean ± SD (*n* = 10), different letters indicate significant differences revealed by Tukey’s test.

**FIGURE 10:**
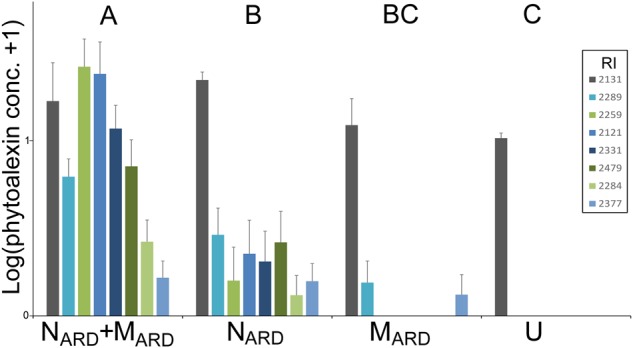
Phytoalexins in roots of apple rootstocks 8 weeks after inoculation with the nematodes and microbes from ARD soil (N_ARD_+M_ARD_), only nematodes from ARD soil (N_ARD_), or only microbes from ARD soil (M_ARD_), and non-inoculated control (U). Mean and SE of log-transformed concentrations (ppm); different letters indicate significant differences revealed by Tukey’s test (*n* = 10) applied to the first principal component from analysis of phytoalexins. The first principle component explained 72.3% of the variance. Compounds that were detected in less than eight samples were omitted from the analysis. Compound retention index (RI) 2131: 2-hydroxy-4-methoxydibenzofuran; 2121: noraucuparin; 2259: noreriobofuran; 2289: isomer of noreriobofuran; 2331: hydroxyeriobofuran; 2479: 3,9-dimethoxy-2,4-dihydroxydibenzofuran; 2284: unknown (MW 404); 2377: hydroxynoreriobofuran.

Concentrations of phenolic compounds in roots were significantly higher in plants treated with the combination of N_ARD_+M_ARD_ compared to that of single inoculations of N_ARD_ or M_ARD_, and compared to that of non-inoculated plants, 4 and 8 weeks after inoculation (Figure 9). Roots inoculated with only N_ARD_ produced significantly more phenolic compounds than the non-inoculated control. In contrast, roots inoculated with only M_ARD_ did not significantly differ from the non-inoculated control, and they showed only 4 weeks but not 8 weeks after inoculation a trend for higher concentrations of total phenolics. For all treatments, lower concentrations of phenolic compounds were detected at the later sampling in this experiment. Determination of phytoalexins in roots 8 weeks after inoculation confirmed a significantly stronger defense response of the plants to N_ARD_+M_ARD_ compared to N_ARD_ or M_ARD_ alone, or compared to non-inoculated plants (Figure 10). Roots inoculated with only N_ARD_ produced significantly more phytoalexins than the non-inoculated control. In contrast, roots inoculated with only M_ARD_ did not significantly differ from the non-inoculated control. The ARD-responsive phytoalexins noraucuparin and noreriobofuran as well as hydroxyeriobofuran and 3,9-dimethoxy-2,4-dihydroxydibenzofuran were only detected in the two treatments with inoculated nematodes from ARD soil. Aucuparin was detected in five samples of these treatments but only in one sample of the treatment with only M_ARD_. The intermediate 2-hydroxy-4-methoxydibenzofuran of the biosynthetic pathway of phytoalexins in apple plants was detected in all treatments, and this was the only compound detected in the non-inoculated control.

## Discussion

A yet unknown complex of soil biota causes ARD (Winkelmann et al., 2019). We investigated the contribution of nematodes to ARD by dissecting the soil biota from soil infested with ARD and non-infested control soil into nematode and microbe fractions, and challenging susceptible M26 apple plants with combinations of these fractions. We investigated whether the microbial community from ARD soil impaired growth of apple plants, and whether nematodes from ARD or control soil added to this effect in a sterile substrate system. We could show in our system that the soil decline leading to symptoms of ARD in apple plants is enhanced by nematodes extracted from the ARD affected soil. Nematodes from ARD soil were required for induction of severe ARD. The microbes synergistically enhanced ARD together with these nematodes. In contrast, microbes alone or nematodes from control soil did not cause such an effect, although ARD microbes when compared to the untreated control significantly impaired apple plant growth.

In this study, differences in species composition between ARD and control soils has been shown, thus giving an indication that the nematode communities associated with the soil decline syndrome leading to ARD may be unique and could be explored further. The highest population of nematodes in the ARD soil were free-living. Probably the shift in nematode community structure in addition to the enrichment of the free-living taxa *Acrobeloides* and *Cephalenchus* could explain the role of non-parasitic nematodes in ARD. *Acrobeloides* is a non-pathogenic bacterivore which is abundant in agricultural soils and associated with a diverse set of microbes (Baquiran et al., 2013). *Cephalenchus* was reported to feed on root cells but is not regarded to cause plant damage (Sutherland, 1967; Siddiqi, 2000). Entomopathogenic *Steinernema* were strongly enriched in the control plots. These are, together with their symbiont bacteria *Xenorhabdus*, obligate and lethal parasites of insects. The effect is probably rather related to the legacy of insects associated with the previous grown grass, than to a protective effect against ARD. The role of free-living nematodes in disease complexes is most often neglected partly because they are bio-indicators for soil health (Mekonen et al., 2017), or because of the overwhelming damage caused by plant parasitic nematodes in general (Jones et al., 2013), which masks their potential effects.

Microscopic inspection of ARD affected roots coupled with molecular approaches revealed that plant-parasitic nematodes were very low in abundance and did not differ significantly between ARD and control soils. We could not detect *P. penetrans* in the diseased roots and the peculiar symptoms of root lesions and lack of feeder roots associated with these nematodes were not observed. These findings support evidence reported by Manici et al. (2018) that plant-parasitic nematodes including the root lesion nematodes do not cause the disease. In their studies using three affected orchard soils, they could not recover any plant-parasitic nematodes from the roots of the affected apple plants. Although Manici et al. (2013) previously reported the presence of root lesion nematodes in affected roots, the low frequency of these nematodes did not give evidence for a contribution to growth reduction in apple.

In our system, typical symptoms associated with ARD, which included stunted growth and reduced root systems were enhanced upon the addition of nematodes extracted from ARD soil to microbes extracted from ARD soil. We could show that nematodes extracted from ARD soil when added to microbes regardless of their origin affected plant growth more severely than treatments without ARD nematodes. The nematodes from ARD soil alone (with the microbiome associated with their bodies or guts) also had significant effects on the plants but less pronounced. Our hypothesis that the addition of ARD nematodes to ARD microbes enhances the disease symptoms is supported by evidence that nematicidal treatments of ARD soil by the moderate heat of 50°C or biofumigation alleviated ARD (Yim et al., 2013, 2016). Harsh treatments like Basamid or heating at 100°C, which targets also microbes, even further reduced ARD symptoms (Yim et al., 2013). Nematodes from the control soil in combination with microbes, either from ARD or control soil, did not induce ARD symptoms. This is in agreement with the observed difference in the nematode species composition between the ARD and control soil, with specific taxa being significantly enriched in the ARD soil. However, further experiments are needed to find out which of the nematode species are involved in the interaction with microbes that leads to the reaction of the roots. The progress or induction of ARD when ARD nematodes were added to ARD microbes started 4 weeks after inoculation and was more evident after 8 weeks of inoculation when ARD nematodes were added to microbes from the control soil. Plants at this later stage were severely affected showing stunted growth. The reduced root systems were brittle and easily break. Mazzola and Manici (2012) observed that ARD symptoms are visible 1–3 months after planting in the field while in a controlled sterile environment, as in our system, symptoms are already visible 2–5 weeks after inoculation. Our studies clearly fit these observations and explain why evident symptoms of the disease start after 4 weeks of inoculation. Depressed plant growth as a result of the addition of ARD nematodes to microbes (ARD or control) manifested in the physiological response of the plant. Plants exposed to ARD nematodes and microbe combinations did not only react with strong growth depression but also with a stress response. It has been previously established that apple plants showing severe growth depression accumulated stress-related compounds or secondary metabolites compared to non-diseased plants (Henfrey et al., 2015). Antioxidants, phytoalexins and phenolic compounds are enhanced in ARD affected roots (Manici et al., 2013; Franke-Whittle et al., 2015) and considerable concentrations of some phenolic compounds have been observed in affected soils as well (Yin et al., 2016). We could show similar effects as ARD nematodes in combination with microbes induced higher concentrations of phenolic compounds than either ARD nematodes or microbes alone. The significant accumulation of stress-related compounds such as phenolics in roots suggests an ongoing defense mechanism within the root (Matern and Grimmig, 1994). Progressively, nematodes alone (with associated microbiome) induced ARD as indicated by the significantly higher concentrations of phenolic compounds compared with non-inoculated control or only ARD microbes. Additionally, plants inoculated with ARD nematodes in combination with ARD microbes produced more phytoalexins compared to only ARD nematodes, microbes or the non-inoculated control. This is consistent with findings of Ahuja et al. (2012) stating that, for ARD affected plants in order to deal with stress imposed by ARD causal agents (Mazzola and Manici, 2012), plants produce high amounts of phytoalexins. The responsive phytoalexins noraucuparin and noreriobofuran as well as hydroxyeriobofuran and 3,9-dimethoxy-2,4-dihydroxydibenzofuran were found in the ARD nematode or with ARD microbe treatments. This supports findings of Weiß et al. (2017) that biotic stress inducing ARD leads to high amounts of phytoalexins in affected roots.

For the first time, the importance of the free-living nematodes and their synergy with soil microbes to induce ARD has been shown in this study. Many disease complexes as a result of nematode-microbe interactions have been reported that focus only on plant-parasitic nematodes (Powell, 1971; Back et al., 2002; Morris et al., 2016). The pathogenicity of bacteria *(Bacillus subtilis*), fungi (*Penicillium janthinellum, Constantinella terrestris, and Trichoderma*) or nematodes (*P. penetrans*) were tested separately or in combinations (Utkhede et al., 1992). They could show that *P. penetrans* alone induced symptoms similar to ARD in apple plants, although in combination with bacteria and/or with fungi the disease was equally induced. In a separate experiment nematodes or microorganisms were recovered from ARD soil or symptomatic apple plants and were assessed for pathogenicity (Mazzola, 1998). Multiple species of the genera *Rhizoctonia, Cylindrocarpon* (or the related genus *Ilyonectria*), and oomycetes (*Pythium, Phytophthora*) were most often associated with ARD. The study also noted that the cited pathogens acted in combination with the lesion nematode *P. penetrans* to enhance the disease severity. The genus *Pythium, Cylindrocarpon*-like species, *Rhizoctonia* and *Fusarium* most consistently coincided with symptoms of ARD (Manici et al., 2013; Franke-Whittle et al., 2015). This was interpreted in a way that multiple biological agents including nematodes are responsible for ARD (Tewoldemedhin et al., 2011). In consequence, many different pathogens would cause the same symptoms. However, a more parsimonious explanation was that secondary infections of ARD affected roots were observed in these studies, and that an interaction involving two or more soil biota and potentially also abiotic factors causes ARD (Winkelmann et al., 2019).

The mechanism underlying the synergy between nematodes and microbes in ARD is yet to be unraveled. However, it is known that a high abundance of nematodes feeding on microbes can modify the microbial community by altering the relative abundance of populations (Djigal et al., 2004; Hai-Feng et al., 2014; Gebremikael et al., 2016), thus causing a significant reduction of microbes that may induce plant growth promotion. Nematodes have a direct or symbiotic association with the soil microbiome and are specifically attached or native to their bodies or gut (Baquiran et al., 2013; Adam et al., 2014; Elhady et al., 2017). Recent findings by Adam et al. (2014) and Elhady et al. (2017) confirmed that specific bacteria and fungi are attached to infective stages of *Meloidogyne incognita* and *P. penetrans* in different soil types, indicating an ecological role of the association. Indirectly, nematodes might contribute to ARD by dissemination of microbes or activation of specific microbial growth by the release of growth-limiting nutrients (Freckman, 1988; Wang et al., 2005). In conclusion, an interaction of specific free-living nematodes with microbes in the rhizosphere of apple plants seems to be essential for the development of ARD. Exploring the associations of nematodes and microbes in ARD soils will give the chance to finally unravel the etiology of ARD.

## Data Availability Statement

The raw data supporting the conclusion of this manuscript will be made available by the authors, without undue reservation, to any qualified researcher. DNA sequencing data are available at NCBI SRA BioProject ID PRJNA497506.

## Author Contributions

HH and XK designed the research, performed the experiments and analyses, and wrote the paper. BL and LB analyzed phytoalexins. SJS contributed NGS data. BL, LB and SJS did discussions and revision of the manuscript.

## Conflict of Interest Statement

The authors declare that the research was conducted in the absence of any commercial or financial relationships that could be construed as a potential conflict of interest.

## Footnotes

1 www.biopieces.org

2 https://www.r-project.org/

## References

[B1] Adam M. Westphal A. Hallmann J. Heuer H. (2014). Specific microbial attachment to root knot nematodes in suppressive soil. *Appl. Environ. Microbiol.* 80 2679–2686. 10.1128/AEM.03905-13 24532076 PMC3993313

[B2] Ahuja I. Kissen R. Bones A. M. (2012). Phytoalexins in defense against pathogens. *Trends Plant Sci.* 17 73–90. 10.1016/j.tplants.2011.11.002 22209038

[B3] Ainsworth E. A. Gillespie K. M. (2007). Estimation of total phenolic content and other oxidation substrates in plant tissues using Folin-Ciocalteu reagent. *Nat. Protoc.* 2 875–877. 10.1038/nprot.2007.102 17446889

[B4] Anderson M. J. (2001). A new method for non-parametric multivariate analysis of variance. *Austral Ecol.* 26 32–46. 10.1111/j.1442-9993.2001.01070.pp.x

[B5] Antweiler K. Schreiter S. Keilwagen J. Baldrian P. Kropf S. Smalla K. (2017). Statistical test for tolerability of effects of an antifungal biocontrol strain on fungal communities in three arable soils. *Microb. Biotechnol.* 10 434–449. 10.1111/1751-7915.12595 28111906 PMC5328832

[B6] Back M. A. Haydock P. P. J. Jenkinson P. (2002). Disease complexes involving plant parasitic nematodes and soilborne pathogens. *Plant Pathol.* 51 683–697. 10.1046/j.1365-3059.2002.00785.x

[B7] Baquiran J.-P. Thater B. Sedky S. Ley P. de Crowley D. Orwin P. M. (2013). Culture-independent investigation of the microbiome associated with the nematode *Acrobeloides maximus*. *PLoS One* 8:e67425. 10.1371/journal.pone.0067425 23894287 PMC3718782

[B8] Barker K. R. (1985). “Nematode extraction and bioassays,” in *An Advanced Treatise on Meloidogyne: Vol. 2 Methodology* eds Barker K. R. Carter C. C. Sasser J. N. (Raleigh, NC: Dept. of Plant Pathology North Carolina State University) 19–35.

[B9] Blaxter M. L. Ley P. de Garey J. R. Liu L. X. Scheldeman P. Vierstraete A. (1998). A molecular evolutionary framework for the phylum Nematoda. *Nature* 392 71–75. 10.1038/32160 9510248

[B10] Cock P. J. A. Grüning B. A. Paszkiewicz K. Pritchard L. (2013). Galaxy tools and workflows for sequence analysis with applications in molecular plant pathology. *PeerJ* 1:e167. 10.7717/peerj.167 24109552 PMC3792188

[B11] Djigal D. Brauman A. Diop T. A. Chotte J. L. Villenave C. (2004). Influence of bacterial-feeding nematodes (Cephalobidae) on soil microbial communities during maize growth. *Soil Biol. Biochem.* 36 323–331. 10.1016/j.soilbio.2003.10.007

[B12] Elhady A. Giné A. Topalovic O. Jacquiod S. Sørensen S. J. Sorribas F. J. (2017). Microbiomes associated with infective stages of root-knot and lesion nematodes in soil. *PLoS One* 12:e0177145. 10.1371/journal.pone.0177145 28472099 PMC5417685

[B13] Erel O. (2004). A novel automated direct measurement method for total antioxidant capacity using a new generation, more stable ABTS radical cation. *Clin. Biochem.* 37 277–285. 10.1016/j.clinbiochem.2003.11.015 15003729

[B14] Franke-Whittle I. H. Manici L. M. Insam H. Stres B. (2015). Rhizosphere bacteria and fungi associated with plant growth in soils of three replanted apple orchards. *Plant Soil* 395 317–333. 10.1007/s11104-015-2562-x

[B15] Freckman D. W. (1988). Bacterivorous nematodes and organic-matter decomposition. *Agric. Ecosyst. Environ.* 24 195–217. 10.1016/0167-8809(88)90066-7 27337962

[B16] Gebremikael M. T. Steel H. Buchan D. (2016). Nematodes enhance plant growth and nutrient uptake under C and N-rich conditions. *Sci. Rep.* 6:32862. 10.1038/srep32862 27605154 PMC5015107

[B17] Hai-Feng X. Gen L. I. Da-Ming L. I. Feng H. U. Hui-Xin L. I. (2014). Effect of different bacterial-feeding nematode species on soil bacterial numbers, activity, and community composition. *Pedosphere* 24 116–124. 10.1016/S1002-0160(13)60086-7

[B18] Henfrey J. L. Baab G. Schmitz M. (2015). Physiological stress responses in apple under replant conditions. *Sci. Hortic.* 194 111–117. 10.1016/j.scienta.2015.07.034

[B19] Hewavitharana S. S. Mazzola M. (2016). Carbon source-dependent effects of anaerobic soil disinfestation on soil microbiome and suppression of *Rhizoctonia solani* AG-5 and *Pratylenchus penetrans*. *Phytopathology* 106 1015–1028. 10.1094/PHYTO-12-15-0329-R 27143411

[B20] Hoestra H. (1994). Ecology and pathology of replant problems. *Acta Hortic.* 363 1–10. 10.17660/ActaHortic.1994.363.1

[B21] Hoestra H. Oostenbrink M. (1962). Nematodes in relation to plant growth. IV. *Pratylenchus penetrans* (Cobb) on orchard trees. *Neth. J. Agric. Sci.* 10 286–296.

[B22] Hooper D. J. Hallmann J. Subbotin S. A. (2005). “Methods for extraction, processing and detection of plant and soil nematodes,” in *Plant Parasitic Nematodes in Subtropical and Tropical Agriculture* eds Luc M. Sikora R. A. Bridge J. (Wallingford: CABI) 53–85. 10.1079/9780851997278.0053

[B23] Hu W. Chen S. Liu X. (2016). Effect of temperature treatment on survival of *Heterodera glycines* and its associated fungi and bacteria. *Nematology* 18 845–855. 10.1163/15685411-00003000

[B24] Hüttner C. Beuerle T. Scharnhop H. Ernst L. Beerhues L. (2010). Differential effect of elicitors on biphenyl and dibenzofuran formation in *Sorbus aucuparia* cell cultures. *J. Agric. Food Chem.* 58 11977–11984. 10.1021/jf1026857 20961041

[B25] Isutsa D. K. Merwin I. A. (2014). Nematodes and fungi associated with apple replant disorder in sampled New York State orchards. *Glob. J. Biosci. Biotechnol.* 3 174–180.

[B26] Jaffee B. A. Abawi G. S. Mai W. F. (1982). Role of soil microflora and *Pratylenchus penetrans* in an apple replant disease. *Phytopathology* 72 247–251. 10.1094/Phyto-72-247

[B27] Jiang J. Song Z. Yang X. Mao Z. Nie X. Guo H. (2017). Microbial community analysis of apple rhizosphere around Bohai Gulf. *Sci. Rep.* 7:8918. 10.1038/s41598-017-08398-9 28827532 PMC5566992

[B28] Jones J. T. Haegeman A. Danchin E. G. J. Gaur H. S. Helder J. Jones M. G. K. (2013). Top 10 plant-parasitic nematodes in molecular plant pathology. *Mol. Plant Pathol.* 14 946–961. 10.1111/mpp.12057 23809086 PMC6638764

[B29] Klaus H. (1939). Das Problem der Bodenmüdigkeit unter Berücksichtigung des Obstbaus. *Landw. Jahrb.* 89 413–459.

[B30] Mahnkopp F. Simon M. Lehndorff E. Pätzold S. Wrede A. Winkelmann T. (2018). Induction and diagnosis of apple replant disease (ARD): a matter of heterogeneous soil properties? *Sci. Hortic.* 241 167–177. 10.1016/j.scienta.2018.06.076

[B31] Mai W. F. Abawi G. S. (1978). Determining the cause and extent of apple, cherry, and pear replant diseases under controlled conditions. *Phytopathology* 68 1540–1544. 10.1094/Phyto-68-1540

[B32] Mai W. F. Abawi G. S. (1981). Controlling replant diseases of pome and stone fruits in Northeastern United States by preplant fumigation. *Plant Dis.* 65 859–864. 10.1094/PD-65-859

[B33] Mai W. F. Merwin I. A. Abawi G. S. (1994). Diagnosis, etiology and management of replant disorders in New York cherry and apple orchards. *Acta Hortic.* 363 34–41. 10.17660/ActaHortic.1994.363.5

[B34] Manici L. M. Caputo F. Saccà M. L. (2017). Secondary metabolites released into the rhizosphere by *Fusarium oxysporum* and Fusarium spp. as underestimated component of nonspecific replant disease. *Plant Soil* 415 85–98. 10.1007/s11104-016-3152-2

[B35] Manici L. M. Kelderer M. Caputo F. Mazzola M. (2015). Auxin-mediated relationships between apple plants and root inhabiting fungi: impact on root pathogens and potentialities of growth-promoting populations. *Plant Pathol.* 64 843–851. 10.1111/ppa.12315

[B36] Manici L. M. Kelderer M. Caputo F. Saccà M. L. Nicoletti F. Topp A. R. (2018). Involvement of *Dactylonectria* and *Ilyonectria* spp. in tree decline affecting multi-generation apple orchards. *Plant Soil* 425 217–230. 10.1007/s11104-018-3571-3

[B37] Manici L. M. Kelderer M. Franke-Whittle I. H. Rühmer T. Baab G. Nicoletti F. (2013). Relationship between root-endophytic microbial communities and replant disease in specialized apple growing areas in Europe. *Appl. Soil Ecol.* 72 207–214. 10.1016/j.apsoil.2013.07.011

[B38] Masella A. P. Bartram A. K. Truszkowski J. M. Brown D. G. Neufeld J. D. (2012). PANDAseq: paired-end assembler for illumina sequences. *BMC Bioinformatics* 13:31. 10.1186/1471-2105-13-31 22333067 PMC3471323

[B39] Matern U. Grimmig B. (1994). Natural phenols as stress metabolites. *Acta Hortic.* 381 448–462. 10.17660/ActaHortic.1994.381.58

[B40] Mazzola M. (1998). Elucidation of the microbial complex having a causal role in the development of apple replant disease in Washington. *Phytopathology* 88 930–938. 10.1094/PHYTO.1998.88.9.930 18944871

[B41] Mazzola M. Hewavitharana S. S. Strauss S. L. (2015). Brassica seed meal soil amendments transform the rhizosphere microbiome and improve apple production through resistance to pathogen reinfestation. *Phytopathology* 105 460–469. 10.1094/PHYTO-09-14-0247-R 25412009

[B42] Mazzola M. Manici L. M. (2012). Apple replant disease: role of microbial ecology in cause and control. *Annu. Rev. Phytopathol.* 50 45–65. 10.1146/annurev-phyto-081211-173005 22559069

[B43] McCune B. Grace J. B. Urban D. L. (2002). *Analysis of Ecological Communities.* Gleneden Beach, OR: MjM software design.

[B44] Mekonen S. Petros I. Hailemariam M. (2017). The role of nematodes in the processes of soil ecology and their use as bioindicators. *Agric. Biol. J. N. Am.* 8 132–140. 10.5251/abjna.2017.8.4.132.140

[B45] Morris K. A. Langston D. B. Dutta B. Davis R. F. Timper P. Noe J. P. (2016). Evidence for a disease complex between *Pythium aphanidermatum* and root-knot nematodes in cucumber. *Plant Health Prog.* 17 200–201. 10.1094/PHP-BR-16-0036

[B46] Nicola L. Insam H. Pertot I. Stres B. (2018). Reanalysis of microbiomes in soils affected by apple replant disease (ARD): old foes and novel suspects lead to the proposal of extended model of disease development. *Appl. Soil Ecol.* 129 24–33. 10.1016/j.apsoil.2018.04.010

[B47] Nicola L. Turco E. Albanese D. Donati C. Thalheimer M. Pindo M. (2017). Fumigation with dazomet modifies soil microbiota in apple orchards affected by replant disease. *Appl. Soil Ecol.* 113 71–79. 10.1016/j.apsoil.2017.02.002

[B48] Oksanen J. Blanchet F. G. Kindt R. Legendre P. Minchin P. R. O’hara R. B. (2015). *vegan: Community Ecology Package. R Package Version 2.3–0. 2015. Google Scholar.*

[B49] Omirou M. Rousidou C. Bekris F. Papadopoulou K. K. Menkissoglou-Spiroudi U. Ehaliotis C. (2011). The impact of biofumigation and chemical fumigation methods on the structure and function of the soil microbial community. *Microb. Ecol.* 61 201–213. 10.1007/s00248-010-9740-4 20811742

[B50] Powell N. T. (1971). Interactions between nematodes and fungi in disease complexes. *Annu. Rev. Phytopathol.* 9 253–274. 10.1146/annurev.py.09.090171.001345

[B51] Quast C. Pruesse E. Yilmaz P. Gerken J. Schweer T. Yarza P. (2013). The SILVA ribosomal RNA gene database project: improved data processing and web-based tools. *Nucleic Acids Res.* 41 D590–D596. 10.1093/nar/gks1219 23193283 PMC3531112

[B52] Riley I. T. Reardon T. B. (1995). Isolation and characterization of *Clavibacter tritici* associated with *Anguina tritici* in wheat from Western Australia. *Plant Pathol.* 44 805–810. 10.1111/j.1365-3059.1995.tb02739.x

[B53] Roberts D. W. (2016). *labdsv: Ordination and Multivariate Analysis for Ecology.* Available at: https://cran.r-project.org/web/packages/labdsv/labdsv.pdf [accessed July 23 2018]

[B54] Robinson M. D. McCarthy D. J. Smyth G. K. (2010). edgeR: a Bioconductor package for differential expression analysis of digital gene expression data. *Bioinformatics* 26 139–140. 10.1093/bioinformatics/btp616 19910308 PMC2796818

[B55] Ross R. G. Crowe A. D. (1973). Replant disease in apple orchard soil. *Can. Plant Dis. Surv.* 53 144–146.

[B56] Siddiqi M. R. (2000). *Tylenchida: Parasites of Plants and Insects.* Wallingford: CABI 10.1079/9780851992020.0000

[B57] Sutherland J. R. (1967). Parasitism of *Tylenchus emarginatus* on conifer seedling roots and some observations on the biology of the nematode. *Nematologica* 13 191–196. 10.1163/187529267X00049

[B58] Tewoldemedhin Y. T. Mazzola M. Labuschagne I. McLeod A. (2011). A multi-phasic approach reveals that apple replant disease is caused by multiple biological agents, with some agents acting synergistically. *Soil Biol. Biochem.* 43 1917–1927. 10.1016/j.soilbio.2011.05.014

[B59] Utkhede R. S. Vrain T. C. Yorston J. M. (1992). Effects of nematodes, fungi and bacteria on the growth of young apple trees grown in apple replant disease soil. *Plant Soil* 139 1–6. 10.1007/BF00012835

[B60] Wang K.-H. McSorley R. Wang K.-H. McSorley V. (2005). *Effects of Soil Ecosystem Management on Nematode Pests, Nutrient Cycling, and Plant Health.* Gainesville, FL: Department of Entomology and Nematology University of Florida 10.1094/APSnetFeatures/2005-0105

[B61] Weiß S. Liu B. Reckwell D. Beerhues L. Winkelmann T. (2017). Impaired defense reactions in apple replant disease-affected roots of *Malus domestica* ’M26’. *Tree Physiol.* 37 1–14. 10.1093/treephys/tpx108 29036594

[B62] Winkelmann T. Smalla K. Amelung W. Baab G. Grunewaldt-Stöcker G. Kanfra X. (2019). Apple replant disease: causes and mitigation strategies. *Curr. Issues Mol. Biol.* 30 89–106. 10.21775/cimb.030.089 30070653

[B63] Yeates G. W. Bongers T. Goede R. G. M. de Freckman D. W. Georgieva S. S. (1993). Feeding habits in soil nematode families and genera - an outline for soil ecologists. *J. Nematol.* 25 315–331. 19279775 PMC2619405

[B64] Yim B. Hanschen F. S. Wrede A. Nitt H. Schreiner M. Smalla K. (2016). Effects of biofumigation using *Brassica juncea* and *Raphanus sativus* in comparison to disinfection using Basamid on apple plant growth and soil microbial communities at three field sites with replant disease. *Plant Soil* 406 389–408. 10.1007/s11104-016-2876-3

[B65] Yim B. Smalla K. Winkelmann T. (2013). Evaluation of apple replant problems based on different soil disinfection treatments — links to soil microbial community structure? *Plant Soil* 366 617–631. 10.1007/s11104-012-1454-6

[B66] Yin C. Xiang L. Wang G. Wang Y. Shen X. Chen X. (2016). How to plant apple trees to reduce replant disease in apple orchard: a study on the phenolic acid of the replanted apple orchard. *PLoS One* 11:e0167347. 10.1371/journal.pone.0167347 27907081 PMC5132267

[B67] Zolla G. Badri D. V. Bakker M. G. Manter D. K. Vivanco J. M. (2013). Soil microbiomes vary in their ability to confer drought tolerance to Arabidopsis. *Appl. Soil Ecol.* 68 1–9. 10.1016/j.apsoil.2013.03.007

