# Supplementary material for: Free-Living Nematodes Together With Associated Microbes Play an Essential Role in Apple Replant Disease
Source: Front Plant Sci. 2018 Nov 16;9:1666. doi: 10.3389/fpls.2018.01666 (PMC6250840; doi:10.3389/fpls.2018.01666)
Supplement: Supplementary file 1 [file Data_Sheet_1.PDF]

Supplement

Free-living nematodes together with associated microbes play an essential role in apple replant disease

Xorla Kanfra[1], Benye Liu[2], Ludger Beerhues[2], Søren J. Sørensen[3], Holger Heuer[1*]

[1] Department of Epidemiology and Pathogen Diagnostics, Julius Kühn-Institut - Federal Research Centre for Cultivated Plants, Braunschweig, Germany

[2] Technische Universität Braunschweig, Institute of Pharmaceutical Biology, Braunschweig, Germany

[3] Section of Microbiology, Department of Biology, University of Copenhagen, Copenhagen, Denmark

*** Correspondence:**

Dr. Heuer Holger

holger.heuer@julius-kuehn.de

Supplementary Table S1. Vegetative growth of apple seedlings in pots inoculated with nematodes from ARD plots (N_{ARD}) and microbes from ARD plots (M_{ARD}), in comparison to the uninoculated control (U). Nematode and microbe communities were extracted from soil of an apple orchard where trees were highly affected by apple replant disease (Heidgraben, Germany). Mean ± SD (n = 10), different letters indicate significant differences revealed by Tukey's test.

**Plant growth parameters**	**Weeks after inoculation**	**N_{ARD}+M_{ARD}**	**M_{ARD}**	**U**
Shoot length [cm]	4	6.9±0.5b	9.2±2.6a	11.4±2.4a
Shoot FM [g]	4	2.3±1.0a	1.9±0.6a	2.6±0.9a
Leaf count [n]	4	17.8±1.8b	18.2±3.5b	12.5±5.1a
Leaf FM [g]	4	1.5±0.6a	1.4±0.4a	1.7±0.5a
Root FM [g]	4	3.2±0.8b	3.5±1.1b	6.2±1.9a
Shoot length [cm]	8	21.1±1.7b	25.1±2.9a	23.9±2.2a
Shoot FM [g]	8	6.8±1.5b	7.2±1.7b	4.5±1.7a
Leaf count [n]	8	21.7±2.3b	25.6±2.1b	14.5±7.1a
Leaf FM [g]	8	3.6±0.6b	5.0±0.6a	3.7±1.2b
Leaf DM [g]	8	1.3±0.4a	1.8±0.5b	1.0±0.3a
Root FM [g]	8	3.1±0.6a	4.6±1.1b	5.8±0.8c

Supplementary Table S2. Vegetative growth of apple seedling in pots inoculated with microbial communities (M), or in addition nematodes (N) extracted from soil of an apple orchard where trees were highly affected by apple replant disease (ARD) and interspersed with control plots (Con), or uninoculated plants (U). Mean ± SD (n = 10), different letters indicate significant differences revealed by Tukey's test.

Plant growth parameter	Weeks after inocula-tion	Inoculants of apple seedlings						
		N_{ARD} + M_{ARD}	N_{ARD} + M_{Con}	N_{Con} + M_{ARD}	N_{Con} + M_{Con}	M_{ARD}	M_{Con}	U
Shoot length [cm]	4	2.8±0.7c	3.5±0.8bc	3.9±0.7abc	4.0±1.1abc	4.6±1.4ab	4.1±1.0abc	5.2±1.3a
Shoot FM [g]	4	1.7±0.4ab	1.8±0.3ab	1.8±0.6ab	1.8±0.3ab	1.9±0.5ab	1.4±0.2b	2.1±0.6a
Leaf count	4	12.2±2.5a	12.8±2.1a	15.1±2.7a	13.2±1.6a	14.7±3.2a	13.3±1.8a	14.1±2.5a
Leaf FM [g]	4	0.9±0.17b	1.0±0.19ab	1.21±0.4ab	1.2±0.2ab	1.4±0.4a	0.9±0.2b	1.2±0.34ab
Root FM [g]	4	2.6±0.7b	2.9±0.8ab	3.6±1.4ab	2.9±0.6ab	2.9±0.9ab	2.6±0.3b	3.8±0.9a
Shoot length [cm]	8	10.6±3.4d	11.3±1.5d	16.6±2.2ab	16.0±2.7ab	16.7±0.8ab	16.4±1.6ab	19.6±3.7a
Shoot FM [g]	8	3.9±0.8b	3.8±0.5b	5.8±0.8a	5.6±1.2a	6.1±0.8a	6.0±0.9a	6.1±0.8a
Leaf count	8	18.7±4.3b	20.3±2.9b	25.1±1.7a	26.5±3.6a	25.4±1.9a	25.2±2.9a	27.6±3.4a
Leaf FM [g]	8	2.3±0.6b	2.4±0.3b	3.7±0.5a	3.5±0.8a	4.1±0.3a	4.1±0.7a	3.6±0.5a
Leaf DM [g]	8	0.4±0.2b	0.4±0.1b	0.9±0.2a	0.8±0.4a	0.90±0.37a	0.80±0.23a	0.90±0.26a
Root FM [g]	8	3.9±0.7c	4.5±1.8c	5.5±2.6bc	6.2±2.6abc	8.5±1.7a	8.9±1.8a	7.9±2.9ab
Root DM [g]	8	0.5±0.2b	0.6±0.4b	1.2±0.7ab	1.2±0.7ab	1.7±0.5a	2.0±0.5a	2.1±1.0a

Supplementary Table S3. Nematode genera/species whose relative abundance increased in apple replant soil as compared to control plots. Data are expressed as mean ± SD. FDR (false discovery rate) < 0.05 was used to denote statistical significance.

**Enriched Taxa (genus/species)**	**ARD**	**Control**	**OTU**	**GenBank Accession No., % Identity**
Cephalenchus	1.71±2.13	0.03±0.06	OTU163	KJ869347.1, 100
Koerneria sp.	0.07±0.11	0±0	OTU68	EU196025.1 , 100
Acrobeloides sp.	80±4.7	39.67±18.43	OTU91	AF034391.1, 100
Cephaloboides nidrosiensis	0.45±0.37	0.22±0.13	OTU95	EU196020.1, 100
Ptycholaimellus sp.	0.17±0.1	0.06±0.1	OTU37	FJ040472.1, 100
Basiria aberrans	0.03±0.05	0±0	OTU153	KJ869318.1, 100
Basiria sp.	0.01±0.01	0±0.01	OTU157	KJ869354.1.,100
Cephalobus cubaensis	0.09±0.11	0.02±0.04	OTU96	AF202161.1, 100

Supplementary Table S4. Mean nematode sequences of individuals in 100 ml soil in the apple replant affected soils (ARD) interspaced with uncultivated grass control soil planted with apple one time (control). Three replicates per block were sampled for both soil types.

	Mean species abundance of individuals ± SD							
	Control				ARD			
Genus	Block A	Block B	Block C	Block D	Block A	Block B	Block C	Block D
Alaimella	0	0	0.3±0.6	0	0	0	0	0
Anaplectus	2794±2401	295.3±189	140±113	436±115	160±82	229±106	132±40	323±236
Camacolaimus	0	0	0	0.7±0.6	0.3±0.6	0.3±0.6	0.3±0.6	1±1
Chronogaster	1±0	1.3±1.5	0	0	0.3±0.6	0	0.3±0.6	0
Cylindrolaimus	0.3±0.6	0.3±0.6	0.7±1.2	0.3±0.6	1.7±1.5	1±1	0.3±0.6	0
Domorganus macronephriticus	5.7±4	0.3±0.6	1.3±0.6	3±4.4	2.7±2.1	0	2±1	0.3±0.6
Hemiplectus muscorum	0	0	0.3±0.6	0	0.7±1.2	0.3±0.6	0	0.3±0.6
Odontophora	0	0	0	0.3±0.6	0	0	0	0.3±0.6
Pakira	5±7	2.3±2.3	0.7±1.2	1.3±1.5	0.7±0.6	0.7±0.6	0.7±1.2	1±1
Paraphanolaimus	0.7±1.2	0	0	0	0	0	0	0
Plectus	68.7±24.1	53.3±36	50.3±39.5	33.3±12	58.3±37.2	93.3±70	74±10.5	123±75.9
Setostephanolaimus	0.3±0.6	0	0	0	0	0	0	0
Tylocephalus	79±89.4	3.7±1.5	106±90	45.3±71.6	5.7±4	8±5.6	6±2.6	10±4.6
Wilsonema	115±39	89±62	541±331	333±332	252±403	29±22	24±11	28±5
Achromadora	188±128	189±74	281±331	195±76	15±15	48±28	123±76	132±170
Choanolaimus	0	0	0	0.3±0.6	0.3±0.6	0	0	0
Halichoanolaimus	0	0	0	0	0.3±0.6	0	0	0.3±0.6
Paracyatholaimus	0	0.3±0.6	0	0.3±0.6	0.7±1.2	0	0	1±0
Prodesmodora	1±1	0	0	0.3±0.6	0	0	0	0.3±0.6
Ptycholaimellus	0.3±0.6	6±4.4	16±26	1.7±2.9	19±10.6	14±14.9	18.3±12.2	40.7±37.5
Spilophorella	0	0.3±0.6	0	0	0.3±0.6	0	0.7±1.2	1±1.7
Acanthopharynx	0.3±0.6	0	0	0	0	0	0	0
Desmodora	0.3±0.6	0	0	0.3±0.6	0.3±0.6	0	0.3±0.6	1.3±1.5
Dracograllus	2.7±2.9	3.7±3.2	2.3±3.2	1.3±1.5	0.7±1.2	2±1	0.7±1.2	5.7±6
Epsilonema	3±3.5	4±3.5	3±4.4	1.3±1.5	0.7±1.2	3.7±2.5	0.7±1.2	6±6
Eubostrichus	33±45.9	57±48.8	31.7±53.1	36±35.7	5±3	48.3±25.5	17.7±26.3	78.7±68.8
Leptonemella	0	0	0	0	0.3±0.6	0	0	0
Paradraconema	0	0	0	0	0.7±1.2	0	0	0
Prodesmodora circulata	1±1.7	0.3±0.6	1±1.7	0	0	1±1	0.7±1.2	0.7±1.2
Robbea	0.3±0.6	0.7±0.6	1.7±2.1	0.7±0.6	0.3±0.6	0	0	0.3±0.6
Robbea	0	0.7±0.6	1.7±2.1	0.7±0.6	0	0	0	0
Stilbonematinae	0	0	0	0	0	0.3±0.6	0	0
Desmoscolex	0	0	0	0	0	0	0.3±0.6	0
Demaniella	0	0	0	0.7±1.2	0	0	0	0.7±0.6
Diplogastridae	0	0	0	0	0	0	0.3±0.6	0
Koerneria	0	0.3±0.6	0.3±0.6	0	4.3±4.5	2.3±2.1	4±6.1	34.3±47.9
Myctolaimus	0	0.3±0.6	0	0	0	0	0	0
Parasitodiplogaster	0	0.7±0.6	0	0	0.7±0.6	0	0	0.3±0.6
Pristionchus	34.3±38.9	1.7±2.1	32.7±14	16.7±4	4.3±3.2	3.3±2.1	2.3±2.1	2±2.6
Rhabditolaimus	0	0	0	0	0	0	0	0.3±0.6
Daptonema	0	0	0	0	0	0	0	0.3±0.6
Desmolaimus	0.3±0.6	0	0	1±1	0	0.7±0.6	0	0.3±0.6
Diplolaimella	0	0	0	0	0.3±0.6	0	0	0
Halomonhystera	0	0	0	0	0.3±0.6	0	0	0
Paralamyctes	2.3±2.5	1.3±0.6	2.3±3.2	0.3±0.6	0.7±0.6	1±1	0.7±0.6	1.3±1.5
Sabatieria	0	0	0	0	0.3±0.6	0	0	0.3±0.6
Terschellingia	0	0	0	0	0	0	0	0.3±0.6
Theristus	0	0	0.3±0.6	0	0	0	0	0
Acrobeles	193±109	233±102	129±94	233±91	198±43	205±53	106±15	210±169
Acrobeloides	7457±1817	2290±951	3301±3011	8033±1630	8799±2600	12650±3566	8954±1029	11608±7580
Alloionema appendiculatum	0	0	0	0	0	0	0	0.3±0.6
Cephaloboides	47.3±23.2	6.7±2.1	25.7±17	17±10.1	26.7±16.1	79.3±56.2	60.7±50	29.7±16.1
Cephalobus	3.3±2.3	0.3±0.6	3.3±1.2	8.3±9.2	12.3±6	6±6.2	22±14.7	3.3±3.2
Cervidellus	26.3±25.4	3±3	22.7±6.5	16.3±18.6	3.3±4.2	1±1	1.3±2.3	2±2.6
Chiloplacus	2.7±2.5	1.7±1.2	0.3±0.6	2.3±0.6	2.3±2.1	2.7±1.5	1.7±0.6	2.7±3.1
Cruznema	3±4.4	1.3±2.3	0	0	2±1.7	0	0	3±2.6

Daubaylia	0.7±1.2	1±1	0	0.3±0.6	1±1	0.3±0.6	1.7±1.5	3.3±4
Dicelis	4.7±5.7	1.7±2.1	0.7±0.6	2.3±0.6	1.3±0.6	1.3±1.2	1.3±0.6	1.3±2.3
Diploscapter	0	0	0	0.3±0.6	0	0	1.3±1.5	0.3±0.6
Drasico	4±3	0.7±1.2	1.7±2.1	2±1	2.3±1.5	2±2.6	2.3±3.2	2.7±1.5
Drilocephalobus	0	0	0	0	0.3±0.6	0	0	0
Eucephalobus	134±72.2	34.3±12.4	64.7±22.3	136.3±78	63.7±24.4	13±3.6	71.3±44.4	91.7±49
Euteratocephalus	0.3±0.6	0	0	0	0	0	0	0
Heterocephalobus elongatus	0.3±0.6	0.3±0.6	0.3±0.6	1±1	0	0	0	0
Heterorhabditis hepialus	0	0	0	1.3±2.3	0	0	0	0
Mesorhabditis	0	0	0	0	0.3±0.6	0	0	0
Metateratocephalus	284±91.8	44.7±30.7	92.3±48.2	73±59.9	20.3±22.3	12±9.6	29.3±22.5	74.7±35
Panagrolaimus	0	0	0	0.3±0.6	0	0	0	0
Pellioditis	0	0	1±0	0	0	0.7±1.2	0.7±1.2	0.3±0.6
Pelodera	9.7±15.9	0.3±0.6	0.3±0.6	0	2.7±2.1	0	168.7±292.1	0
Plectonchus	0	0	0	0.3±0.6	0	0	0	0
Poikilolaimus	1.3±0.6	0	0.3±0.6	0.7±1.2	0.3±0.6	1.3±0.6	0	0
Pseudacrobeles	87.3±37.2	60±61.7	22.3±12.7	42.7±18.9	65±56.7	8.3±2.3	23.3±6.1	55.3±24.2
Rhabditis	7.3±10.1	4.7±8.1	0.7±1.2	2.3±1.2	0	2.7±3.1	0	0.7±1.2
Rhabditoides	0.3±0.6	0	0	0.3±0.6	0	0	0	0
Rhabditophanes	364±513.5	0	7±2.6	8±6.1	0	0.7±1.2	0	0
Seleborca	9±11.4	272.3±207.2	5±5.2	1±1.7	0.3±0.6	2.7±2.3	0	0
Steinernema	488±519	2374±2590	3072±1122	1038±1225	7.3±6.7	3.7±1.5	1.3±1.5	63±106
Teratocephalus	0.3±0.6	0	0.3±0.6	0.3±0.6	0	0	0	0.3±0.6
Zeldia	1±0	1±1	0.7±0.6	1.7±2.1	2.3±2.3	3±2	1.7±1.5	0.3±0.6
Heth	0	0.3±0.6	0.7±1.2	0	0	0	0	0
Xystrognathus	0	0	0	0	0	0	0.3±0.6	0
Trophomera	1±1.7	0	0	0	0	0	0.3±0.6	0
Aglenchus	214.7±150.3	26±10.6	69±51.6	83.3±49.8	125.3±41.4	356±404.5	52±27.5	117.7±29.4
Amplimerlinius	1±1.7	0.3±0.6	1.3±1.5	0.3±0.6	0	2.3±4	0.3±0.6	0.7±1.2
Anguina	0.3±0.6	0	0	0	0	0	0	0
Anurida	0	0	0	0	0	0	0.3±0.6	0
Aphelenchus	1±1	0	0.3±0.6	0	0.3±0.6	0.7±1.2	1±1	0.7±1.2
Basiria	7±4.6	5.3±1.5	5.7±2.5	7.7±1.5	19.7±7.4	8±7.9	7±1.7	16±8.7
Belonolaimus	0.3±0.6	0	0	0	0	0	0	0
Bitylenchus	39.7±49	80±55.6	0.3±0.6	14.3±22.3	1.3±1.2	3±4.4	0.3±0.6	1±1
Boleodorus	2±2	0.7±1.2	3±3.5	1.7±2.1	2.3±2.1	2.7±4.6	2.7±2.5	0.3±0.6
Carphodorus	0	0	0	0	0	0	0.3±0.6	0
Cephalenchus	3±1	1±1	1±1.7	7.3±11.8	172.3±87	686.3±834.5	36.3±27.6	107.3±60.5
Coslenchus	82.3±68.2	12.3±4.2	33.7±23	13±7	34±18.2	40.3±35.2	21.7±11.2	32.3±23.1
Deladenus	1033.7±1748.1	0.3±0.6	78.3±106	167±187.6	0	0.3±0.6	1±1.7	0.3±0.6
Ditylenchus	39.3±22	7±6.6	23.7±27.1	20±28.6	32.7±14.4	30.3±45.6	78±14.2	38.7±23.4
Ecphyadophora	0	0	0.7±1.2	0	0.3±0.6	0	3.7±4.6	1.3±2.3
Fergusobia	93±157.6	0	7±6.6	8.7±12.5	0	0	0	0
Filenchus	11±3.5	2.3±3.2	4.3±7.5	2±1	0	0.3±0.6	0.3±0.6	2±1
Halenchus	0	0	0	0	0.7±1.2	0	0	0
Helicotylenchus	0	4.3±4.5	0.3±0.6	0.3±0.6	0	0	0	0
Hemicaloosia	0	0	0	0.3±0.6	0	0	0	0
Hemicycliophora	0.3±0.6	0	0	0	0.3±0.6	0	0	0
Heterodera	0	6±7.9	1±1	0	0	0	0.3±0.6	0
Hexatylus	0	0	0	0.3±0.6	0	0	0	0
Hirschmanniella	1±1	0.7±0.6	0	0	0.7±0.6	0.3±0.6	0.7±0.6	1±1
Hoplolaimus	0.3±0.6	1±1	0	0	0	0.7±1.2	0	0.7±1.2
Howardula	1.7±2.9	0	0	0	0	0.3±0.6	0	0
Irantylenchus vicinus	0.3±0.6	0	0	0	0	0	0	0
Macrotrophurus	0	0	0	0	0.3±0.6	0	0	0
Malenchus	0	0	0.3±0.6	0	0	0	0	0
Meloidogyne	0	0	0	5.7±9.8	0.3±0.6	0	0	0.7±0.6
Mesocriconema	1.3±0.6	0	0	0	0	0	0	0
Neodolichorhynchus	0.3±0.6	0	0	0	0.3±0.6	0	0	0.7±0.6
Neopsilenchus	60.3±32.3	8±6	15.7±9.1	11.3±4.2	3.7±4	7.3±3.8	3.7±2.9	9.7±8.1
Parasitylenchus bifurcatus	0.3±0.6	0	0	0	0.3±0.6	0	0	0
Paratylenchus	23±24.3	520.3±602.4	18±27.7	27.7±45.3	3±2.6	2.3±1.5	5.3±3.2	3.3±0.6
Pratylenchus	3.3±4.9	29.3±24.2	4.7±7.2	47.7±37.2	19.7±2.3	68.7±76.2	76.7±65.5	23±23.6
Pseudhalenchus	47±56.7	1.3±2.3	0.3±0.6	2±1.7	0.7±0.6	2.3±3.2	1.7±2.1	11±8.5
Psilenchus	1±1	0	0	0	1±0	0.3±0.6	1.3±1.5	0.3±0.6
Rotylenchus	0.3±0.6	0.3±0.6	225±252	7±6.1	0	0	0	0

Sphaerularia	0.3±0.6	0	0	0	0	0.7±1.2	0.3±0.6	0
Subanguina	0.3±0.6	0.3±0.6	0	0.3±0.6	0.3±0.6	1±1	0.3±0.6	0
Telotylenchus	1±1	0.7±1.2	0.3±0.6	0.7±0.6	0.3±0.6	1.3±1.5	0	0.3±0.6
Trophurus	0	0	0	0	0	0	0.3±0.6	0
Tylenchocriconema	3±2.6	0	1.3±1.5	0	0	0	2.3±1.5	0.3±0.6
Tylenchorhynchus	1±1	1.3±1.2	0	0.7±0.6	1±1	0.3±0.6	0.7±0.6	0
Tylenchus	7±11.3	2±2	0.3±0.6	0	2±1	0	1.3±1.5	4.3±3.5
Vittatidera	0	0	0.3±0.6	0	0	0	0	0
Zygotylenchus	0	0	0.3±0.6	0	0.3±0.6	0	0	0
**Sum**	**42,444**	**20265**	**25392**	**33480**	**30515**	**44099**	**30473**	**39964**
